# Around the Hospitals

**Published:** 1894-12-01

**Authors:** 


### Around the Hospitals.

[Notice to the Managers of Institutions.—Special space is now reserved for the insertion of notices of hospital meetings and festivals. The Editor requests that all notices may reach The Hospital Office, 428, Strand, at least one week before the date of each meeting.]

**The Adelaide Hospital, Dublin.**—This hospital is a Protestant religious foundation receiving general and fever cases. The rules and regulations are not modern in spirit, and no monthly or quarterly courts appear to be held for the conduct of the business of the hospital. The uniform system of accounts is not adopted, and the cost per patient or occupied bed is not given in the report. Paying patients are received into private wards and pay beds. In 1892 the hospital received a bequest of £2,000 for effecting improvements and repairs. This sum has wisely been devoted to securing separate accommodation for fever nurses, besides other additions to the nurses' quarters. The system of baths and lavatories in connection with the fever wards has been also remodelled. Further sanitary improvements are also suggested. The Fetherston-Haugh Convalescent Home in connection with the hospital is now completed, and will accommodate twelve male and twelve female patients. It will thus be seen that much advance in the completeness of the institution has been made. The report of the visitors from the Dublin Hospital Sunday Fund was to the effect that all was in good order, clean, comfortable, well ventilated, and heated. During the past year 1,208 cases were treated. The ordinary income amounted to £6,799, the expenditure to £6,943.

**The Seamen's Hospital, Greenwich.**—The sphere of work undertaken by the Seamen's Hospital Society, which has its head-quarters at the hospital, Greenwich, is constantly increasing, and there is scope for further development at Tilbury when funds are forthcoming. As it is the offer of the Dock Company to provide a building at Tilbury for hospital purposes cannot be accepted at present. Last year the income exceeded the expenditure largely, and a curtailment even of present work threatens unless the society be better supported. The large number of 17,749 sick and injured sailors were treated during 1893. The cases admitted are mostly serious, being frequently of long standing, and of an aggravated nature, owing to the circumstances attending a sailor's calling. Many of the patients who are disabled or destitute, or both, are, on leaving the hospital, helped through the society. The General Steam Navigation Company and the Shipwrecked Fishermen and Mariners' Royal Benevolent Society co-operate in this part of the work. The crowned heads of most nations contribute towards the hospital, it being recognised that sailors of all nationalities are cared for there. The support of the public is asked not only on account of what is done for mariners, but because no accident cases of a serious nature are refused, be the patient landsman or sailor. The organisations of the Seamen's Society consist of, besides the hospital at Greenwich, one at the Victoria and Albert Docks, and of dispensaries at the East India Docks and at Gravesend. So scattered an area of work is difficult of administration, and all credit should be given to the successful efforts of the management. Patients during the past year were distributed thus: At Greenwich, 2,254 in-patients and 5,672 out-patients; at the Victoria and Albert Docks, 328 in, and 5,299 out-patients; at the dispensaries 3,220 patients attended at Wells Street and 974 at Gravesend. During 1893 the ordinary income amounted to £12,194, and the expenditure to £14,860. There is therefore a deficiency of £2,204 on the year's accounts. The cost per occupied bed on the ordinary expenditure amounted to £69 8s. 10d. Only one shilling is deducted for each out-patient, which is, according to usual experience, too small a sum to represent the expenditure under this head. Most hospitals show a desire to over and not under estimate the expenses of the out-patient department.

